# Invasion Potential of Two Tropical *Physalis* Species in Arid and Semi-Arid Climates: Effect of Water-Salinity Stress and Soil Types on Growth and Fecundity

**DOI:** 10.1371/journal.pone.0164369

**Published:** 2016-10-14

**Authors:** Cumali Ozaslan, Shahid Farooq, Huseyin Onen, Bekir Bukun, Selcuk Ozcan, Hikmet Gunal

**Affiliations:** 1 Department of Plant Protection, Dicle University, Diyarbakir, Turkey; 2 Department of Plant Protection, Gaziosmanpaşa University, Tokat, Turkey; 3 Pisachio Research Station, Gaziantep, Turkey; 4 Department of Soil Science and Plant Nutrition, Gaziosmanpaşa University, Tokat, Turkey; University of Wyoming, UNITED STATES

## Abstract

Invasive plants are recognized for their impressive abilities to withstand adverse environmental conditions however, all invaders do not express the similar abilities. Therefore, survival, growth, nutrient uptake and fecundity of two co-occurring, invasive *Physalis* species were tested under water and salinity stresses, and different soil textures in the current study. Five different water stress levels (100, 75, 50, 25, and 12.5% pot water contents), four different soil salinity levels (0, 3, 6, and 12 dSm^-1^) and four different soil textures (67% clay, 50% clay, silt clay loam and sandy loam) were included in three different pot experiments. Both weeds survived under all levels of water stress except 12.5% water contents and on all soil types however, behaved differently under increasing salinity. The weeds responded similarly to salinity up till 3 dSm^-1^ whereas, *P*. *philadelphica* survived for longer time than *P*. *angulata* under remaining salinity regimes. Water and salinity stress hampered the growth and fecundity of both weeds while, soil textures had slight effect. Both weeds preferred clay textured soils for better growth and nutrient uptake however, interactive effect of weeds and soil textures was non-significant. *P*. *angulata* accumulated higher K and Na while *P*. *philadelphica* accrued more Ca and Mg as well as maintained better K/Na ratio. *P*. *angulata* accumulated more Na and P under salinity stress while, *P*. *philadelphica* accrued higher K and Mg, and maintained higher K/Na ratio. Collectively, highest nutrient accumulation was observed under stress free conditions and on clay textured soils. *P*. *philadelphica* exhibited higher reproductive output under all experimental conditions than *P*. *angulata*. It is predicted that *P*. *philadelphica* will be more problematic under optimal water supply and high salinity while *P*. *angulata* can better adapt water limited environments. The results indicate that both weeds have considerable potential to further expand their ranges in semi-arid regions of Turkey.

## Introduction

Invasive plants pose significant threats to native biodiversity, human and animal health, agricultural production and disrupt ecosystem services throughout the world [1]. The higher tolerance, survival and growth of invasive plants under moisture deficiency, high soil salinity, and shade along with continuous disturbances enable them to successfully survive and spread in introduced ranges [2, 3, 4]. However, all of the invasive plants don't exhibit similar growth potential and tolerance to harsh environments [5]. Therefore, a better knowledge regarding growth potential of invasive plants under adverse environmental conditions will help to predict their potential distribution ranges, identify the most detrimental invasive plants and devising management tools to stop their further range expansion.

Habitat conditions and available resources regulate occurrence and spread of invasive plants in introduced ranges [6]. Soil moisture is the main ecological indicator among habitat resources [7, 8] and water stress is considered as major factor responsible for shaping plant communities [9] especially in arid and semi-arid areas. Climate forecasts suggest that severe drought events will be observed globally in the coming decades [10]. The increasing water scarcity due to climate change will negatively affect the survival and growth of plants, substantially leading to lower ecosystem productivity [11, 12]. Water is the crucial driver of ecosystem services, and its deficiency will alter nutrient cycles resulting in decreased nutrient uptake due to low available moisture in the soil [13, 14]. The agricultural practices such as irrigation, to cope with water deficiency have been increasing soil salinity [15, 16] in arid and semi-arid regions.

Plant community structures are highly fragile and greatly affected by edaphic and environmental factors such as water availability and soil salinity [17, 18]. However, research relating to plant invasion on saline or sodic soils is sparse, though it appears that these sites can act as edaphic refuges for native species [19]. Besides, different soil types offer varying water and nutrient availability because of surface area variations for nutrient and water absorptions. Among different soil textures, clay provide a higher surface area for nutrient absorption and water holding [20].

Nutrient and moisture uptake determine the growth performance of plant species under stressed and benign environments [11]. According to invasion resistance hypothesis, drought and salinity are important barriers to the establishment and spread of invasive plants in the introduced ranges [21]. Since drought and salinity occur simultaneously in arid and semi-arid regions, both factors offer hurdles in the establishment and spread of invasive plants. However, invasive plants may develop adaptation strategies to survive under stressed conditions which help them to dominate in new habitats [22].

The diverse topography and varying elevations throughout the country presents a great diversity of climate in Turkey. There exists an extreme variation in year to year rainfall in Mediterranean climate [23] and plants often experience cyclic or prolonged episodes of drought stress. It is also predicted that future climate warming will further worsen the situation [24]. The South Eastern part and Central Anatolia regions of Turkey are characterized by arid and semi-arid climates with hot summers according to Thornthwaite climate classification. The hot summers result in high evapotranspiration, and water demands of the plants are fulfilled by irrigation. Irrigation along with high evaporation leads to soil salinization which negatively affects plant growth and development. Invasive plants become more competitive than natives under increasing aridity and salinity due to better adaptation abilities [25]. Successful management of invasive plants is often constrained by the lack of knowledge regarding their environmental requirements for seedling recruitment, survival and reproduction [8]. Hence, identification of environmental regimes which promote or limit invasion could be helpful for managing the spread and establishment of invasive plants. Although the adaptations of invasive plants are considered as key to successful invasions, experimental studies of actual adaptive responses under different abiotic stresses and soil types have rarely been tested.

Two groundcherry species, *Physalis angulata* L. (cutleaf groundcherry, Solanaceae) and *Physalis philadelphica* Lam. var. immaculata Waterfall (Mexican groundcherry) have been reported as invaders and weeds in several parts of the world. *P*. *angulata* has been reported as a noxious weed of several crops such as rice, cotton and soybean [26], and an invasive plant in several countries of the world [27]. *P*. *angulata* was firstly reported as an invasive plant in Turkey in 2000 [28], while *P*. *philadelphica* was reported in 2002 [29]. After their first introduction, both plants rapidly expanded their distribution range in the country and became troublesome weeds of several crops [26, 28]. Both weeds have mainly been reported in the tropical and sub-tropical climatic regions of the world [30], and their presence in arid and semi-arid regions makes them a matter of special concern. Climate forecasts suggest increased aridity and salinity in the arid and semi-arid regions of the country [24]. Therefore, effects of ecological changes on distribution of *P*. *angulata* and *P*. *philadelphica* and their adaptation strategies under ecological changes are also subjects of increased interest.

The current study was therefore planned to; i) infer survival, biomass production, nutrient uptake and reproduction of co-occurring *P*. *angulata* and *P*. *philadelphica* under water and salinity stress and different soil textures ii) investigate adaptation potentials and strategies to cope with adverse environmental conditions and, iii) determine optimal moisture and salinity ranges for their growth and development to incorporate the results in predicting potential distribution ranges. The results will help to model their potential distribution ranges and identify the strategies to cope with the adverse environmental conditions.

## Materials and Methods

### Experimental setup

Three different pot experiments were conducted in greenhouse located at Gaziosmanpaşa University, Tokat, Turkey (40.33°N, 36.47°E, 640 m asl). The greenhouse was maintained at 28/22±5°C day/night temperatures and 16 h photoperiod throughout the experimental period. Water stress, salinity stress and soil type treatments were considered as separate experiments. All experiments (water stress, salinity stress and soil type) were laid out in a randomized complete block design with split plot arrangements. For a given experiment, (i.e., water stress, soil salinity or soil type) invasive weeds were kept in the main plots while treatments were randomized into sub-plots. Each experimental treatment had five replications and repeated over time (two experimental runs for each kind of experiment).

The experiments were conducted in free draining plastic pots (8.8 liter) filled with 2.7 kg soil (different soil textures according to the experiments and treatments). The physical and chemical characteristics of the soils used in different experiments are represented in Table 1. The soils were collected from the Kazova Plain in Tokat province. Pots were supplied with 200 mg kg^-1^ N, 100 mg kg^-1^ P, 125 mg kg^-1^ K, 2.5 mg kg^-1^ Zn and 2.5 mg kg^-1^ Fe at the beginning of the experiments. Three seedlings were transplanted, and reduced to one per pot 10 days after transplanting (DAT) by keeping the uniform and vigorously growing seedlings at the start of stress treatments. All experiments were harvested at 80 DAT.

**Table 1:** Physical and chemical characteristics of different types of soils used in water stress, salinity and soil types experiments.

Experiments	Chemical properties				Physical properties				
	pH	EC	CaCO_3_	P	OM*	Clay	Sand	Silt	Texture Class
		dSm^-1^	%	mg kg^-1^	%	%	%	%	
**Water stress and salinity**	8.38	0.33	13.5	7.49	1.47	48.2	35.0	16.8	Clay
**Soil types**	8.05	0.31	14.6	8.40	1.15	67.7	12.3	20.0	Clay**
	7.58	0.85	8.1	47.31	2.58	50.2	14.8	35.0	Clay^†^
	7.76	0.17	7.5	11.76	1.54	32.7	19.8	47.5	Silty Clay Loam
	7.54	0.28	5.8	20.60	4.17	32.7	47.3	20.0	Sandy Loam

* = Organic matter

** = clay-1 type soil

^†^ = clay-2 type soil

### Water stress experiment

Seedling survival, growth, nutrient uptake and reproduction were tested under 5 different water stress levels. Water stress levels were determined based on the % pot water contents (PWCs). The water stress treatments were; 100% (control/no stress), 75% (mild stress), 50% (moderate stress), 25% (high stress) and 12.5% (severe stress) PWCs. Stress intensities were determined based on the frequently reported intensities of water stress in literature [31, 32, 33, 34].

The PWCs were measured before initiating the experiment. For measuring PWCs, pots were filled with soil and irrigated until water started to drain from the bottom of the pots. Pots were covered with polyethene sheets and allowed to drain out extra amount of water for 24 hours. Pots were weighed after 24 hours and the amount of water absorbed by soil was taken as 100% PWCs [35]. The pots were maintained at their respective water contents (i.e., 100, 75, 50, 25 0r 12.5%) from initiation of stress treatments to harvest The pots were weighed daily to maintain PWCs, and the amount of evaporated and transpired water was supplied to each pot. This practice was continued until the harvest.

### Salinity stress experiment

In the second experiment, survival, growth, nutrient uptake and fecundity of the weeds were tested under four salinity levels. The soil salinity levels included were; 0 (control/no salinity), 3 (moderate salinity), 6 (high salinity) and 12 dSm^-1^ (severe salinity). Soil salinity levels were achieved by applying NaCl solution of known concentration to the pots [36]. Salinity was slowly raised to avoid the sudden injury to plants. Salinity levels were achieved in one week and afterwards, pots were irrigated with distilled water throughout the experiment.

### Soil types experiment

Survival, growth, nutrient acquisition and reproductive output were tested on four different soil textures, i.e. [clay-1 (67.7% clay), clay-2 (50.2% clay), silty clay loam, and sandy loam] in the third experiment. The clay particles provide higher surface area for nutrient adsorption and water holding. Therefore, two soils with different clay content were included in the study. Pots were irrigated daily according to the moisture requirements of plants to avoid the negative effects of water stress on plant growth.

### Plat material and seed germination

Seeds of tested weeds were collected from a highly infested cotton field in Diyarbakir province of Turkey during 2013 (41.85°N, 37.61°E). The mature and healthy fruits were collected from >50 plants and brought to laboratory. Fruits were dried under shade for 2 weeks and seeds were separated. Seeds were dried and stored in glass jars until use. Dormancy was released by placing the seeds under running tap water for 24 hours. The seeds were germinated in an incubators at 30°C and seedlings were shifted to 72 celled plastic trays filled with potting mix. Seedlings were watered daily and low strength Hoagland solution was applied to the trays. After one week, uniform and healthy seedlings were transplanted to pots.

### Observations

#### Seedling survival time and survival percentage

Seedlings of all experiments were monitored daily after transplanting till harvest for recording seedling survival time and survival percentage. Seedlings with rigorous mortality signs (yellowing, wilting, drying etc.) were harvested immediately. Seedling survival time was taken as days from the initiation of stress treatment to harvest. Moreover, growth and nutrient uptake observations for each of the dying seedling were determined at harvest. The survival percentage was calculated by the following equation;

$$\mathrm{Survival}\%=\frac{\mathrm{Number of surviving seedlings}}{\mathrm{Number of surviving seedlings}}\times 100$$

#### Growth and fecundity traits

The surviving seedlings were harvested at 80 DAT and different growth and fecundity traits were observed. Plant height (PH) was measured with a meter rod from first node to the tip of the top leaf for surviving plants and averaged. The mature fruits produced by each plant were counted to measure the reproductive output. Mature plants were taken off the pots with intensive care to avoid any damage to the roots. Plants were separated into above and below ground parts by cutting from near to soil surface. Potting soil was thoroughly washed to obtain roots and root length (RL) was measured. Above and below ground parts were weighed fresh on an electronic balance and dried in an oven at 65 ± 5°C for 72 hours. Dry weights of above and below ground parts were then taken. Total fresh mass (TFM) and total dry mass (TDM) was calculated by adding the fresh and dry weights of above and below ground parts. Fresh and dry mass ratio (FDR) was computed by dividing TFM with TDM. Relative growth rate (RGR) was calculated by the following equation [37];

$$RGR=\frac{\text{W2-W1}}{\text{T2-T1}}$$

Here; W_2_ is seedling weight at harvest, W_1_ is seedling weight at stress initiation (for drought and salinity) or start of the experiment (for soil types experiment), T_2_ is time of harvest (DAT) and T_1_ is time of stress initiation or start of the experiment.

The biomass allocation to shoots and roots was calculated by dividing the shoot or root dry weight to total dry weight of the plants and expressed as percentage. Biomass allocation to shoot was regarded as shoot mass fraction (SMF) while, to roots was named as root mass fraction (RMF). Root-to-shoot ratio (RSR) was computed by dividing root dry weight to shoot dry weight. Before drying the plants, fruits were separated and dried under shade to separate the seeds for assessing reproductive output. The seeds were manually separated (20 fruits from each plant) and carefully counted. The number of counted seeds were averaged to get number of seeds per fruit. The number of seeds per fruit was multiplied with the total number of fruits to get number of seeds per plant. In case the number of fruits was less than 20, number of seeds in all fruits were counted.

#### Nutrient uptake/accumulation

Nutrient uptake under water and salinity stress, and different soil textures was determined from the aerial parts (collectively from stem and leaves) only. The above ground biomass was rinsed with deionized water and dried in an oven. The dried plant materials were ground to powder. A pre-weighed quantity of the powder was burnt in a microwave oven to get the ash. The ash was digested in 2 ml of 35% H_2_O_2_ and 5 ml of 65% HNO_3_. Following the digestions, potassium (K), calcium (Ca), magnesium (Mg) and sodium (Na) were analyzed on an atomic absorption spectrophotometer (AAS, Agilent 24 FS) [38]. Phosphorus (P) concentration was recorded by the Barton method [39]. Nutrient uptake values were converted to mg g^-1^ of dry mass. The K/Na ratio was calculated by dividing the K and Na concentrations.

#### Statistical Analysis

The statistical analysis of the collected data for growth and nutrient uptake was performed in four different steps for each of experiment separately. Firstly, normality in the data was tested by Shapiro–Wilk test, and variables like RGR and FDR in water stress experiment, RSR and P uptake in salinity experiment and RGR, RSR and Na uptake in soil texture experiment had non-normal distributions. These variables were normalized by log transformation. Secondly, the differences between experimental runs for each experiment were tested by using the paired t-test. Due to non-significant differences between experimental runs, data of the two experimental runs were combined thus, the finally analyzed data had 10 replications. Thirdly, two-way analysis of variance (ANOVA) was used to compute the differences of measured response variables (separately for each type of experiment) among weeds, experimental treatments, and interactions between treatments and invasive weeds. Following two-way ANOVA for each type of experiment, means were grouped by using least significant difference test (LSD) at 95% probability level. Finally, correlation between growth and nutrient uptake traits was tested by using Spearman correlation. Although linear trend was observed in fresh and dry biomass production in water stress and salinity experiments, trend in nutrient uptake was non-linear. The non-linear trend was also recorded in soil texture experiment, thus Spearman correlation was preferred over Pearson correlation. All the statistical computations were performed on SPSS statistical software version 21.0 [40].

## Results

### Water stress experiment

#### Seedling survival

**Fig 1:**
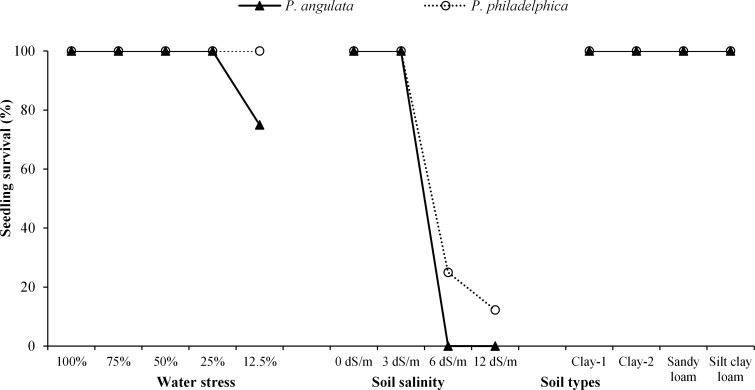
Seedling survival (%) of two co-occurring invasive weeds grown under water and salinity stress and soil types (n = 10)

The invasive weeds responded similarly to different intensities of water stress for seedling survival except severe stress. *P*. *philadelphica* survived for longer period compared to *P*. *angulata* under severe water stress (Table 2). Mortality was observed for some seedlings of *P*. *angulata* (25% mortality) under severe stress whereas, all seedlings of *P*. *philadelphica* were able to survive under all levels of water stress (Fig 1).

**Table 2:** Seedling survival time (days) and survival % of two co-occurring invasive weeds grown on different soil types, and under water and salinity stress.

Water stress experiment			Salinity experiment			Soil types experiment		
Pot water contents	W_1_	W_2_	Salinity levels	W_1_	W_2_	Soil types	W_1_	W_2_	
**100%**	80	80	**0 dSm**^**-1**^	80	80	**Clay-1**	80	80	
**75%**	80	80	**3 dSm**^**-1**^	80	80	**Clay-2**	80	80	
**50%**	80	80	**6 dSm**^**-1**^	19.25	45.25	**Silty Clay Loam**	80	80	
**25%**	80	80	**12 dSm**^**-1**^	12.25	20.35	**Sandy Loam**	80	80	
**12.5%**	65.25	80							

W_1_ = *Physalis angulata*, W_2_ = *Physalis philadelphica*

#### Growth and fecundity

**Fig 2:**
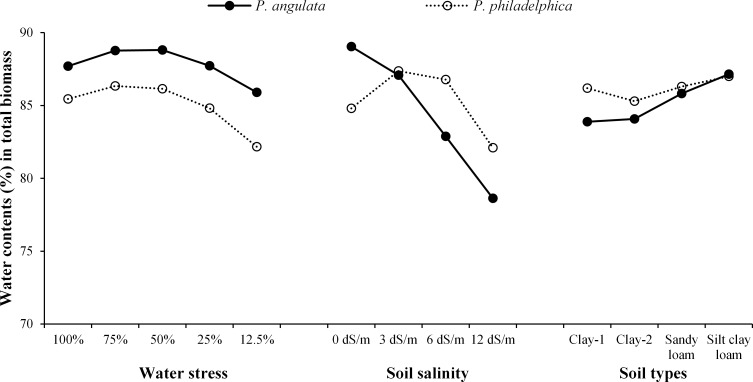
Water contents (%) in total dry mass (± standard error of means) of two co-occurring invasive weeds grown under water and salinity stress, and different soil types (n = 10).

There were no significant differences among both weeds for all the observed growth traits except TFM and FDR (Table 3). *P*. *angulata* had higher TFM and DFR compared with *P*. *philadelphica* (Table 4). Different water stress treatments significantly differed for growth traits (Table 3). The RGR, PH, TFM, TDM, FDR, SMF and RL were higher under control treatment, while severe water stress resulted in abrupt decline in growth traits (Table 4). Contrastingly, highest RMF and RSR were observed under high and severe water stress treatments. Among tested weeds, *P*. *angulata* consumed higher amount water as compared to *P*. *philadelphica* under all water stress treatments (Fig 2).

**Table 3:** Analysis of variance for growth traits of two co-occurring invasive weeds under drought and salinity stresses and soil types.

	Drought stress				Salinity stress				Soil types			
	DF	SS	F	P	DF	SS	F	P	DF	SS	F	P
**Relative growth rate (g plant**^**-1**^ **initial weight**^**-1**^ **day**^**-1**^**)**												
W	1	0.0004	0.20	0.734^ns^	1	0.008	2.29	0.136^ns^	1	0.014	8.30	0.006^‡^
T	4	0.650	757.51	0.000^†^	3	0.289	26.93	0.000^†^	3	0.098	19.70	0.000^†^
W×T	4	0.007	8.37	0.000^†^	3	0.076	7.05	0.000^†^	3	0.014	2.82	0.047*
**Plant height (cm)**												
W	1	38.8	0.07	0.832^ns^	1	7686	140.35	0.000^†^	1	7999	106.59	0.000^†^
T	4	50954.1	727.41	0.000^†^	3	23613	143.72	0.000^†^	3	1486	6.60	0.001**
W×T	4	259.9	3.71	0.008^‡^	3	4001.1	24.35	0.000^†^	3	46.15	0.20	0.893^ns^
**Total fresh mass (g plant**^**-1**^**)**												
W	1	2967.2	4578.7	0.009^‡^	1	1973.0	10.19	0.002^‡^	1	9147	26.89	0.000^†^
T	4	148069	919.68	0.000^†^	3	85195	146.69	0.000^†^	3	10772	10.55	0.000^†^
W×T	4	592.3	3.67	0.009^‡^	3	2117	3.65	0.018*	3	8758	8.58	0.000^†^
**Total dry mass (g plant**^**-1**^**)**												
W	1	1.46	0.16	0.758^ns^	1	86.15	20.14	0.000^†^	1	67.22	8.30	0.006^‡^
T	4	2523.2	732.34	0.000^†^	3	1492.2	116.30	0.000^†^	3	478.53	19.70	0.000^†^
W×T	4	13.44	3.90	0.006^‡^	3	151.99	11.85	0.000^†^	3	68.60	2.82	0.047*
**Fresh mass:dry mass ratio**												
W	1	51.45	3.22	0.001*	1	1.99	0.83	0.367^ns^	1	1.71	1.71	0.196^ns^
T	4	25.75	8.24	0.000^†^	3	82.42	11.43	0.000^†^	3	14.11	4.72	0.005^‡^
W×T	4	0.609	0.19	0.000^†^	3	34.87	4.83	0.005^‡^	3	3.06	1.02	0.389^ns^
**Shoot mass fraction (% shoot plant**^**-1**^**)**												
W	1	0.009	0.46	0.621^ns^	1	0.011	2.58	0.114^ns^	1	0.004	3.37	0.072^ns^
T	4	0.121	10.18	0.000^†^	3	0.442	35.80	0.000^†^	3	0.043	12.61	0.000^†^
W×T	4	0067	5.68	0.000^†^	3	0.034	2.77	0.050*	3	0.007	2.01	0.123^ns^
**Root length (cm)**												
W	1	13.73	0.12	0.785^ns^	1	86.96	3.72	0.059^ns^	1	207.4	7.84	0.007^‡^
T	4	3934.04	45.89	0.000^†^	3	12738	181.88	0.000^†^	3	692.6	8.73	0.000^†^
W×T	4	309.44	3.61	0.100^ns^	3	265.70	3.79	0.015*	3	573.1	7.23	0.000^†^
**Root mass fraction (% root plant**^**-1**^**)**												
W	1	0.009	0.46	0.620^ns^	1	0.011	2.58	0.114^ns^	1	0.004	3.37	0.072^ns^
T	4	0.122	10.26	0.000^†^	3	0.442	35.80	0.000^†^	3	0.043	12.61	0.000^†^
W×T	4	0.063	5.63	0.000^†^	3	0.034	2.77	0.050*	3	0.007	2.01	0.123^ns^
**Root:shoot ratio**												
W	1	0.040	0.43	0.631^ns^	1	0.058	4.04	0.049*	1	0.010	3.88	0.054^ns^
T	4	0.542	8.64	0.000^†^	3	1.142	26.30	0.000^†^	3	0.096	12.79	0.000^†^
W×T	4	0.282	450	0.002^‡^	3	0.122	2.80	0.048*	3	0.014	1.87	0.145^ns^

W = weeds, T = treatment (either pot water contents, or soil salinity levels, or soil types), W×T = interactions among weeds and treatments of different experiments, RGR = relative growth rate, PH = plant height, TFM = total fresh mass, TDM = total dry mass, FDR = fresh mass:dry mass ratio, SMF = shoot mass fraction, RL = root length, RMF = root mass fraction, RSR = root:shoot ratio

^†^ = significant at *p* ≤ 0.0001

** = significant at *p* ≤ 0.001

^‡^ = significant at *p* ≤ 0.01

^ns^ = non-significant

* = significant at *p* ≤ 0.05.

**Table 4:** Effect of different drought intensities on growth and nutrient uptake traits of two co-occurring invasive weeds.

	Growth traits									Nutrient uptake traits					
	RGR	PH	TFM	TDM	FDR	SMF	RL	RMF	RSR	K	Mg	Na	Ca	P	K/Na
	(g g^-1^ day^-1^)	(cm)	(g plant^-1^)	(g plant^-1^)		(% shoot)	(cm)	(% root)		(mg/g)	(mg/g)	(mg/g)	(mg/g)	(mg/g)	
**W**_**1**_	0.11	53.83	60.99 a	7.20	8.37 a	73.0	30.08	27.0	0.38	59.9 a	6.51 b	2.32 a	5.92 b	1.96	27.52 b
**W**_**2**_	0.11	55.22	48.80 b	6.92	6.76 b	75.0	29.25	25.0	0.34	44.8 b	7.04 a	0.61 b	6.23 a	1.88	74.71 a
**LSD 5%**	**NS**	**NS**	**3.11**	**NS**	**0.457**	**NS**	**NS**	**NS**	**NS**	**0.29**	**0.027**	**0.13**	**0.19**	**NS**	**3.81**
**F**_**1**_	0.25 a	87.11 a	117.4 a	15.6 a	7.55 ab	78.0 a	39.66 a	22.0 b	0.28 b	45.2 b	6.63	1.63 a	5.91 c	2.23 a	46.76 bc
**F**_**2**_	0.17 b	73.29 b	87.95 b	10.9 b	8.12 ab	78.0 a	35.16 b	22.0 b	0.29 b	54.4 a	7.17	1.84 a	6.80 a	2.12 ab	42.08 c
**F**_**3**_	0.10 c	60.47 c	51.13 c	6.27 c	8.16 a	76.0 a	28.55 c	24.0 b	0.32 b	57.3 a	6.82	1.42 b	5.72 c	1.91 b	58.76 a
**F**_**4**_	0.02 d	32.69 d	12.81 d	1.69 d	7.40 b	69.0 b	24.84 d	31.0 a	0.47 a	56.4 a	6.72	1.21 b	5.73 c	1.53 c	59.35 a
**F**_**5**_	0.01 e	19.05 e	5.20 e	0.78 e	6.61c	71.0 b	20.11 e	29.0 a	0.46 a	48.6 b	6.71	1.40 b	6.22 b	1.40 c	48.66 b
**LSD 5%**	**0.011**	**3.53**	**4.93**	**0.69**	**0.72**	**0.04**	**3.37**	**0.04**	**0.09**	**0.47**	**NS**	**0.21**	**0.31**	**0.20**	**6.03**
**W**_**1**_**F**_**1**_	0.24 b	82.91 b	126.3 a	15.54 a	8.21 ab	76.0 abc	37.78 ab	24.0 bcd	0.33 cd	50.8 cd	6.11 b	2.72 a	5.71 cd	1.81 c	19.04 g
**W**_**2**_**F**_**1**_	0.26 a	91.31 a	108.4 b	15.78 a	6.87 c	81.0 a	41.53 a	19.0 d	0.23 d	39.5 f	7.03 a	0.51 d	6.01 bc	2.52 a	74.47 bc
**W**_**1**_**F**_**2**_	0.16 d	73.50 c	92.14 c	10.35 c	8.92 a	79.0 ab	35.71 b	21.0 cd	0.29 d	58.1 b	7.24 a	2.74 a	6.42 b	2.18 b	22.47 fg
**W**_**2**_**F**_**2**_	0.18 c	73.07 c	83.75 d	11.44 b	7.32 bc	78.0 ab	34.60 b	22.0 cd	0.27 d	50.7 cd	7.08 a	0.82 d	7.12 a	2.13 b	61.68 d
**W**_**1**_**F**_**3**_	0.10 e	60.09 d	60.80 e	6.80 d	9.11 a	77.0 ab	28.90 c	23.0 cd	0.29 d	66.8 a	7.21 a	2.23 b	6.32 b	2.11 b	30.70 ef
**W**_**2**_**F**_**3**_	0.09 e	60.85 d	41.46 f	5.74 e	7.21 bc	73.0 bc	28.20 cd	27.0 bc	0.35 cd	47.7 de	6.31 b	0.53 d	5.01 e	1.72 c	86.81 a
**W**_**1**_**F**_**4**_	0.03 f	32.66 e	17.52 g	2.15 f	8.20 ab	70.0 cd	23.96 d	30.0 ab	0.43 bc	68.1 a	6.40 b	1.82 c	5.62 cd	1.83 c	38.13 e
**W**_**2**_**F**_**4**_	0.01 fg	32.72 e	8.104 h	1.23 fg	6.60 cd	67.0 d	25.72 cd	33.0 a	0.49 ab	44.6 def	7.01 a	0.52 d	5.81 cd	1.11 d	80.55 ab
**W**_**1**_**F**_**5**_	0.02 fg	19.96 f	8.09 h	1.14 g	7.40 bc	65.0 d	24.04 d	35.0 a	0.58 a	56.0 bc	6.00 b	2.15 bc	5.52 d	1.6 0c	27.29 fg
**W**_**2**_**F**_**5**_	0.001 g	18.13 f	2.30 h	0.41 g	5.80 d	77.0 ab	16.17 e	23.0 cd	0.32 cd	41.2 ef	7.30 a	0.60 d	6.93 a	1.22 d	70.03 cd
**LSD 5%**	**0.015**	**5.00**	**6.97**	**0.98**	**1.02**	**0.52**	**4.76**	**0.54**	**0.13**	**6.61**	**0.62**	**0.31**	**0.44**	**0.28**	**8.52**

NS = non-significant, W1 = Physalis angulata, W2 = Physalis philadelphica, F1 = 100% pot water contents, F2 = 75% pot water contents, F3 = 50% pot water contents, F4 = 25% pot water contents, F5 = 12.5 pot water contents, RGR = relative growth rate, PH = plant height, TFM = total fresh mass, TDM = total dry mass, FDR = fresh mass:dry mass ratio, SMF = shoot mass fraction, RL = root length, RMF = root mass fraction, RSR = root:shoot ratio, K = potassium, Mg = magnesium, Na = sodium, Ca = calcium, P = phosphorus, K/Na = potassium:sodium ratio, The values following different letters in a column are significantly different from each other at p ≤ 0.05

Regarding interactions between water stress treatments and tested weeds, *P*. *philadelphica* observed minimum and maximum RGR under no and severe water stress treatments, respectively. Similarly, *P*. *philadelphica* produced taller plants (91.31 cm) under stress free conditions while, the lowest PH (19.96 and 18.13 cm) for both weeds was recorded under severe water stress (Table 4). *P*. *angulata* produced higher fresh biomass (TFM) than *P*. *philadelphica* under all watering treatments. However, both weeds had similar TDM under no, high and severe stress conditions, while behaved differently under mild and moderate stress (Table 4). The highest FDR was recorded for *P*. *angulata* under all watering treatments. Both weeds allocated higher proportion of biomass to shoots under lower moisture deficit while, more biomass was assimilated to roots under higher intensity of water stress with slight differences between tested weeds (Table 4). Root length of both weeds was similar under all watering treatments except severe stress, and *P*. *angulata* produced longer roots (24.04 cm) than *P*. *philadelphica* (16.17 cm). No significant differences were recorded for RSR between the weeds except under severe water stress (Table 3) where, *P*. *angulata* had higher RSR (0.58) compared to *P*. *philadelphica* (0.32) (Table 4).

#### Nutrient uptake

There were significant differences (*p*≤0.05) in nutrient uptake between both weeds (Table 5). *P*. *angulata* accumulated higher K and Na while better uptake of Mg and Ca was noted for *P*. *philadelphica*. There was no significant difference in P uptake between the weeds however, *P*. *philadelphica* maintained higher K/Na ratio than *P*. *angulata* (Table 4). Increasing intensity of water stress significantly affected the nutrient uptake except Mg. The highest K was accrued under mild, moderate and high water stress while, the lowest was observed under no and severe water stress conditions (Table 4). The highest Ca uptake was noted under mild stress, followed by severe water stress. P uptake was linearly decreased with increasing severity of water stress (Table 4). The highest K/Na ratio was observed under moderate and severe water stress treatments (Table 4).

**Table 5:** Analysis of variance for nutrient uptake traits of two co-occurring invasive weeds under drought and salinity stresses and soil types.

	Drought stress				Salinity stress				Soil types			
	DF	SS	F	P	DF	SS	F	P	DF	SS	F	P
**Potassium (%)**												
W	1	45.76	4.23	0.000^†^	1	4.93	7.20	0.010^‡^	1	0.23	0.39	0.536^ns^
T	4	17.61	17.24	0.000^†^	3	32.16	15.6	0.000^†^	3	26.39	15.05	0.000^†^
W×T	4	6.43	6.30	0.000^†^	3	28.24	13.7	0.000^†^	3	2.11	1.21	0.316^ns^
**Magnesium (%)**												
W	1	0.030	1.35	0.004^‡^	1	0.047	5.80	0.019**	1	0.229	8.10	0.006^‡^
T	4	0.028	2.13	0.086^ns^	3	0.113	4.62	0.006^‡^	3	1.723	20.35	0.000^†^
W×T	4	0.126	9.33	0.000^†^	3	0.157	6.42	0.001*	3	0.121	1.43	0.243^ns^
**Sodium (%)**												
W	1	0.547	39.0	0.000^†^	1	54.59	88.37	0.000^†^	1	9.32	262.7	0.000^†^
T	4	0.032	13.33	0.000^†^	3	353.2	190.6	0.000^†^	3	0.27	2.54	0.066^ns^
W×T	4	0.019	7.96	0.000^†^	3	33.86	18.27	0.000^†^	3	0.31	2.95	0.041**
**Calcium (%)**												
W	1	0.013	49.0	0.006^‡^	1	0.042	2.69	0.106^ns^		0.005	1.64	0.206^ns^
T	4	0.138	17.29	0.000^†^	3	4.418	94.5	0.000^†^	1	0.272	29.3	0.000^†^
W×T	4	0.174	21.83	0.000^†^	3	0.809	17.3	0.000^†^	3	0.014	1.52	0.220^ns^
**Phosphorus (%)**												
W	1	0.002	0.13	0.778^ns^	1	0.012	9.29	0.004^‡^	1	0.068	42.16	0.000^†^
T	4	0.083	31.92	0.000^†^	3	0.007	1.85	0.149^ns^	3	0.064	13.39	0.000^†^
W×T	4	0.045	17.51	0.000^†^	3	0.004	0.93	0.430^ns^	3	0.007	1.42	0.246^ns^
**Potassium:sodium ratio**												
W	1	44529	2470.8	0.000^†^	1	4137	128.4	0.000^†^	1	56544	1777.4	0.000^†^
T	4	3724.4	12.47	0.000^†^	3	34719	359.3	0.000^†^	3	1126	11.80	0.000^†^
W×T	4	1015.2	3.40	0.013*	3	8991	93.05	0.000^†^	3	1207	12.65	0.000^†^

W = weeds, T = treatment (either pot water contents, soil salinity levels, or soil types), W×T = interactions among plants and applied treatments in different experiments, K = potassium, Mg = magnesium, Na = sodium, Ca = calcium, P = phosphorus, K/Na = potassium:sodium ratio

^†^ = significant at *p* ≤ 0.0001

* = significant at *p* ≤ 0.001

^‡^ = significant at *p* ≤ 0.01

** = significant at *p* ≤ 0.05

^ns^ = non-significant.

In interactions among weeds and water stress intensities, *P*. *angulata* accumulated higher amounts of K and Na under all treatments compared with *P*. *philadelphica*. Whereas, better Mg and Ca uptake was noted for *P*. *philadelphica* compared to *P*. *angulata* (Table 4). Better accumulation of P was noted for *P*. *philadelphica* under stress free conditions whereas increased P uptake was noted in *P*. *angulata* with increasing water stress. *P*. *philadelphica* maintained the highest K/Na ratio under all watering treatments compared to its co-occurring *P*. *angulata* (Table 4).

**Fig 3:**
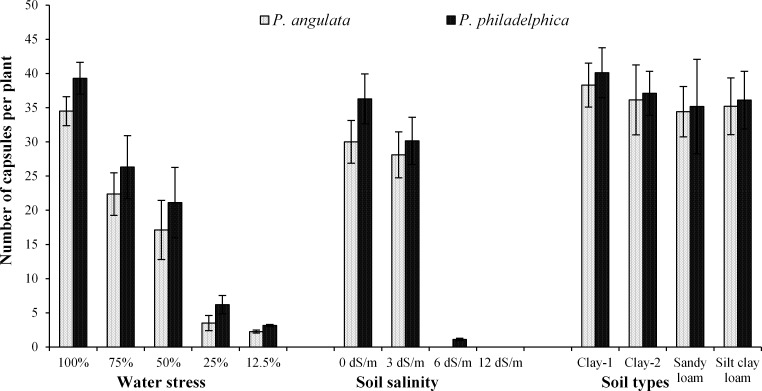
Number of seed bearing fruits (± standard error of means) produced by two co-occurring invasive weeds grown under water and salinity stress and different soil types (n = 10).

**Fig 4:**
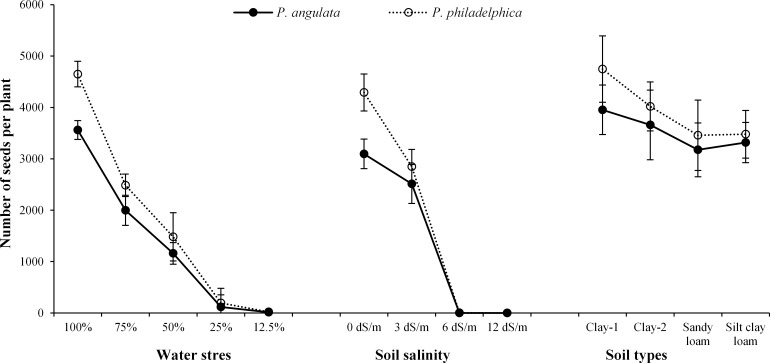
Number of seed per plant (± standard error of means) produced by two co-occurring invasive weeds grown under water and salinity stress and different soil types (n = 10).

Reproductive output of both weeds was decreased with increasing water stress and the lowest reproductive output (19 seeds per plant) was noted under severe water stress (Figs 3 and 4). Among tested weeds, *P*. *philadelphica* exhibited higher reproductive output compared with *P*. *angulata* under all water stress treatments.

Different growth and nutrient uptake traits had significant positive and negative correlations (*p*≤0.05 or 0.01). The RGR was positively correlated with PH, TFM, TDM, SMF, RL and P while had negative correlations with RMF and RSR. TFM and TDM had similar positive correlations as of RGR, with an additional positive correlation by Na. Phosphorus had positive correlations with all observed growth and nutrient uptake traits except RMF, RSR and Mg (Table 6). Interestingly, Na was positively correlated with TFM, TDM, FDR, and K accumulation. However, K had the only positive correlation with FDR. Calcium was positively and negatively correlated with SMF and Mg, and RMF and RSR, respectively (Table 6).

**Table 6:** Correlation between growth and nutrient uptake traits of two co-occurring invasive weeds grown under water stress.

	RGR	PH	TFM	TDM	FDR	SMF	RL	RMF	RSR	K	Mg	Na	Ca	P	K/Na
**RGR**	1														
**PH**	.950**	1													
**TFM**	.971**	.948**	1												
**TDM**	.998**	.944**	.979**	1											
**FDR**	.179	.225*	.354**	.209	1										
**SMF**	.452**	.530**	.472**	.450**	.214	1									
**RL**	.817**	.849**	.827**	.818**	.256*	.375**	1								
**RMF**	-.452**	-.530**	-.472**	-.450**	-.214	-1.00**	-.375**	1							
**RSR**	-.444**	-.518**	-.460**	-.441**	-.186	-.986**	-.358**	.986**	1						
**K**	-.060	-.058	.064	-.035	.730**	-.001	.000	.001	.008	1					
**Mg**	.014	.126	.016	.003	-.017	.405**	.031	-.405**	-.382**	.008	1				
**Na**	.187	.158	.348**	.232*	.715**	.077	.207	-.077	-.054	.673**	-.054	1			
**Ca**	.021	.050	.022	.013	.007	.352**	.061	-.352**	-.336**	-.037	.574**	.048	1		
**P**	.664**	.682**	.673**	.658**	.447**	.576**	.603**	-.576**	-.562**	.382**	.207	.311**	.268*	1	
**K/Na**	-.212	-.146	-.353**	-.256*	-.561**	-.021	-.196	.021	-.003	-.434**	.117	-.931**	-.086	-.192	1

* = Correlation is significant at *p* ≤ 0.05

** = Correlation is significant at *p* ≤ 0.01

RGR = relative growth rate, PH = plant height, TFM = total fresh mass, TDM = total dry mass, FDR = fresh mass:dry mass ratio, SMF = shoot mass fraction, RL = root length, RMF = root mass fraction, RSR = root:shoot ratio, K = potassium, Mg = magnesium, Na = sodium, Ca = calcium, P = phosphorus, K/Na = potassium:sodium ratio.

### Soil salinity experiment

#### Seedling survival

Increasing soil salinity negatively affected the seedling survival of both weeds. However, higher seedling survival percentage and survival time were observed for *P*. *philadelphica* compared with *P*. *angulata* under all salinity treatments similar to water stress experiment (Fig 1; Table 2).

#### Growth and fecundity

The tested weeds exhibited non-significant differences in growth traits except PH, TFM, TDM and RSR (Table 3). *P*. *philadelphica* had higher PH, TFM and TDM than *P*. *angulata* while, better RSR was observed for *P*. *angulata* (Table 7). Different salinity levels significantly affected growth traits of both weeds (Table 3). Increasing salinity linearly reduced PH, TFM, TDM, FDR, RL, RMF and RSR while, SMF of both weeds was increased (Table 7). The higher proportion of biomass was allocated to shoots (SMF) under high and severe salinity levels compared with no and moderate salinity (Table 7). RGR was linearly decreased up to high salinity level and then increased. The increase in RGR is probably due the lesser seedling survival time under severe salinity level (Table 7). Water uptake had interesting results with increasing salinity. Like water stress experiment, more water consumption was recorded for *P*. *angulata* under control treatment, while, more water uptake was recorded for *P*. *philadelphica* with increasing salinity (Fig 2).

**Table 7:** Effect of different soil salinity levels on growth and nutrient uptake of two co-occurring invasive weeds.

	Growth traits									Nutrient uptake traits					
	RGR	PH	TFM	TDM	FDR	SMF	RL	RMF	RSR	K	Mg	Na	Ca	P	K/Na
	(g g^-1^ day^-1^)	(cm)	(g plant^-1^)	(g plant^-1^)		(% shoot)	(cm)	(% root)		(mg/g)	(mg/g)	(mg/g)	(mg/g)	(mg/g)	
**W**_**1**_	0.085	21.60 b	29.09 b	3.47 b	7.31	79.1	24.53	20.9	0.29 a	50.7 b	7.31 b	39.1 a	10.2	2.1 a	7.72 b
**W**_**2**_	0.108	43.52 a	40.19 a	5.79 a	6.95	82.9	22.19	17.1	0.23 b	56.3 a	7.83 a	20.6 b	10.7	1.8 b	23.80 a
**LSD 5%**	**NS**	**3.70**	**6.96**	**1.03**	**NS**	**NS**	**NS**	**NS**	**0.06**	**4.1**	**0.41**	**3.9**	**NS**	**0.18**	**2.84**
**S**_**1**_	0.204 a	61.63 a	92.75 a	12.41 a	7.97 a	71.2 b	39.37 a	28.8 a	0.40 a	64.9 a	6.9 b	1.7 d	6.4 c	1.8	56.04 a
**S**_**2**_	0.079 b	37.86 b	38.62 b	4.92 b	8.37 a	73.1 b	35.35 b	26.9 a	0.39 a	50.8 bc	7.9 a	17.1 c	9.7 b	1.9	4.46 b
**S**_**3**_	0.019 c	17.88 c	5.22 c	0.78 c	6.72 b	87.3 a	10.79 c	12.7 b	0.16 b	45.6 c	7.9 a	35.9 b	13.0 a	2.0	1.58 b
**S**_**4**_	0.084 b	12.89 c	1.98 c	0.40 c	5.47 c	90.4 a	7.93 c	9.6 b	0.10 b	52.7 b	7.6 a	64.7 a	12.5 a	2.1	0.97 b
**LSD 5%**	**0.042**	**5.24**	**9.85**	**1.46**	**1.09**	**0.45**	**3.42**	**0.44**	**0.08**	**0.58**	**0.6**	**5.5**	**0.8**	**NS**	**4.01**
**W**_**1**_**S**_**1**_	0.143 b	39.41 c	79.52 b	8.71 b	9.27 a	70.4 d	42.99 a	29.6 a	0.43 a	73.2 a	7.2 bc	2.7 e	7.3 f	2.0	27.52 b
**W**_**2**_**S**_**1**_	0.266 a	83.85 a	105.98 a	16.11 a	6.67 cd	73.2 cd	35.75 bc	26.8 ab	0.36 ab	56.6 bc	6.5 c	0.7 e	5.5 g	1.6	84.55 a
**W**_**1**_**S**_**2**_	0.061 cde	23.48 d	29.65 d	3.83 d	8.74 ab	68.0 d	37.98 b	32.0 a	0.49 a	41.6 d	7.2 bc	23.6 c	9.0 e	1.9	1.84 cd
**W**_**2**_**S**_**2**_	0.098 bcd	52.24 b	47.58 c	6.01 c	7.99 abc	77.9 c	32.73 c	22.1 b	0.29 bc	60.0 b	8.7 a	10.6 d	10.4 d	1.8	7.07 c
**W**_**1**_**S**_**3**_	0.021 e	12.88 e	4.85 e	0.83 e	6.16 de	86.7 b	9.36 d	13.3 c	0.17 cd	41.6 d	7.1 bc	44.4 b	11.1 cd	2.1	0.95 d
**W**_**2**_**S**_**3**_	0.018 e	22.88 d	5.60 e	0.74 e	7.28 bcd	87.2 ab	12.21 d	12.8 cd	0.15 d	49.6 cd	8.7 a	27.4 c	14.9 a	1.8	2.20 cd
**W**_**1**_**S**_**4**_	0.117 bc	10.65 e	2.34 e	0.50 e	5.07 e	91.8 a	7.77 d	9.2 d	0.08 d	46.4 d	7.7 b	85.6 a	13.2 b	2.2	0.56 d
**W**_**2**_**S**_**4**_	0.050 de	15.13 e	1.62 e	0.29 e	5.87 de	90.0 ab	8.09 d	10.0 cd	0.12 d	58.9 b	7.5 b	43.8 b	11.9 c	1.9	1.37 d
**LSD 5%**	**0.059**	**7.41**	**13.93**	**2.07**	**1.55**	**0.64**	**4.83**	**0.65**	**0.12**	**8.2**	**0.9**	**7.8**	**1.2**	**NS**	**5.68**

NS = non-significant, W_1_ = *Physalis angulata*, W_2_ = *Physalis philadelphica*, S_1_ = no salinity, S_2_ = 3 dSm^-1^ soil salinity, S_3_ = 6 dSm^-1^ soil salinity, S_4_ = 12 dSm^-1^ soil salinity, RGR = relative growth rate, PH = plant height, TFM = total fresh mass, TDM = total dry mass, FDR = fresh mass:dry mass ratio, SMF = shoot mass fraction, RL = root length, RMF = root mass fraction, RSR = root:shoot ratio, K = potassium, Mg = magnesium, Na = sodium, Ca = calcium, P = phosphorus, K/Na = potassium:sodium ratio, The values following different letters in a column are significantly different from each other at *p* ≤ 0.05

Regarding interactions among tested weeds and salinity levels, *P*. *philadelphica* had better RGR, PH, TFM,TDM, SMF under no and moderate salinity levels, while both weeds performed almost similar under higher salinity levels. In contrast, *P*. *angulata* had higher RL, RMF and RSR under control and moderate soil salinity whereas, both weeds performed similar under higher levels of salinity (Table 7).

#### Nutrient uptake

Salinity significantly affected nutrient uptake of both weeds (Table 5). *P*. *philadelphica* accumulated higher amounts of K and Mg while, better uptake of Na and P was noted for *P*. *angulata* (Table 7). There was no significant difference among both weeds for Ca uptake except in interactive effect of weeds and salinity levels (Table 5). A higher K/Na ratio was recorded for *P*. *philadelphica*. Potassium uptake was decreased up to high salinity and increased under severe salinity level. Whereas a steep decline in K/Na ratio was noted with increasing salinity. The Na, Mg and Ca accumulations were increased with rising salinity levels (Table 7).

Regarding interactions among tested weeds and salinity levels, *P*. *philadelphica* accumulated higher amounts of K with increasing salinity. Both tested weeds acquired similar amounts of Mg under no and severe salinity levels. However, under moderate and high salinity treatments, better accumulation of Mg was observed for *P*. *philadelphica* compared with *P*. *angulata*. *P*. *angulata* accumulated almost double amount of Na compared with *P*. *philadelphica*. Highest K/Na ratio was maintained by *P*. *philadelphica* under all salinity levels compared to *P*. *angulata* (Table 7). Increasing salinity suppressed the reproductive output of both weeds. However, *P*. *philadelphica* exhibited higher reproductive potential in comparison to *P*. *angulata* under all salinity levels (Figs 1 and 2). *P*. *philadelphica* even produced a little quantity of seeds (3 seeds per plant) under high salinity while *P*. *angulata* was unable to produce any seed under high salinity (Figs 3 and 4).

Growth and nutrient uptake attributes were positively and negatively correlated with each other as observed in water stress experiment (Table 8). RGR was positively correlated with PH, TFM, TDM, RL, RMF, RSR, K and K/Na ratio, while had negative correlations with SMF, Na and Ca (Table 8). Plant height, TFM and TDM had positive correlations with each other and RL, RSR, K, and K/Na ratio whereas, negatively correlated with Na and Ca. Interestingly, P was negatively correlated with PH. Sodium and Ca had negative correlations with all growth and nutrient uptake traits except SMF and Mg (Table 8). The Na was also positively correlated with P accumulation. K/Na ratio was positively correlated with all growth and nutrient acquisition traits except SMF, Na, Ca, and P where it had negative correlations while, exhibited no correlation with Mg (Table 8).

**Table 8:** Correlation between growth and nutrient uptake traits of two co-occurring invasive weeds grown under different soil salinity levels.

	RGR	PH	TFM	TDM	FDR	SMF	RL	RMF	RSR	K	Mg	Na	Ca	P	K/Na
**RGR**	1														
**PH**	.624**	1													
**TFM**	.743**	.870**	1												
**TDM**	.776**	.853**	.986**	1											
**FDR**	.060	.469**	.501**	.404**	1										
**SMF**	-.481**	-.619**	-.755**	-.752**	-.527**	1									
**RL**	.489**	.752**	.817**	.810**	.577**	-.768**	1								
**RMF**	.481**	.619**	.755**	.752**	.527**	-1.00**	.768**	1							
**RSR**	.481**	.619**	.755**	.752**	.527**	-1.00**	.768**	1.00**	1						
**K**	.392**	.506**	.330**	.291*	.296*	-.215	.217	.215	.215	1					
**Mg**	-.205	-.056	-.229	-.227	-.060	.224	-.242	-.224	-.224	.070	1				
**Na**	-.573**	-.918**	-.882**	-.874**	-.504**	.707**	-.793**	-.707**	-.707**	-.470**	.115	1			
**Ca**	-.653**	-.685**	-.830**	-.823**	-.405**	.737**	-.736**	-.737**	-.737**	-.316*	.551**	.739**	1		
**P**	-.038	-.268*	-.171	-.175	-.162	.193	-.266*	-.193	-.193	.158	.130	.281*	.077	1	
**K/Na**	.588**	.918**	.840**	.824**	.497**	-.669**	.748**	.669**	.669**	.591**	-.105	-.979**	-.725**	-.255*	1

* = Correlation is significant at *p* ≤ 0.05

** = Correlation is significant at *p* ≤ 0.01

RGR = relative growth rate, PH = plant height, TFM = total fresh mass, TDM = total dry mass, FDR = fresh mass:dry mass ratio, SMF = shoot mass fraction, RL = root length, RMF = root mass fraction, RSR = root:shoot ratio, K = potassium, Mg = magnesium, Na = sodium, Ca = calcium, P = phosphorus, K/Na = potassium:sodium ratio.

### Soil types experiment

#### Seedling survival

Different soil textures had no effect on seedling survival of tested weeds. All the transplanted seedlings survived until harvest (Fig 1, Table 2).

#### Growth and fecundity

Tested weeds significantly differed for growth traits except FDR, SMF, RMF and RSR (Table 3). *P*. *philadelphica* observed better growth traits in comparison with *P*. *angulata*. Different soil textures significantly affected measured growth traits (Table 3). The plants grown on clay textured soils had the highest RGR, PH, TFM, TDM, RL, RMF and RSR, while plants grown on sandy loam remained poor in this regard (Table 9). A higher FDR was observed on silt clay loam and sandy loam soils (Table 9). The highest water uptake was noted for *P*. *philadelphica* under all soil textures compared with *P*. *angulata* (Fig 2).

**Table 9:** Growth and nutrient uptake of two co-occurring invasive weeds grown on different soil types.

	Growth traits									Nutrient uptake traits					
	RGR	PH	TFM	TDM	FDR	SMF	RL	RMF	RSR	K	Mg	Na	Ca	P	K/Na
	(g g^-1^ day^-1^)	(cm)	(g plant^-1^)	(g plant^-1^)		(% shoot)	(cm)	(% root)		(mg/g)	(mg/g)	(mg/g)	(mg/g)	(mg/g)	
**W**_**1**_	0.286 b	110.05 b	136.65 b	20.16 b	6.96	83.0	33.62 b	17.0	0.21	38.2	10.2 a	8.3 a	4.7	1.9 b	4.82 b
**W**_**2**_	0.315 a	132.41 a	160.56 a	22.21 a	7.28	85.0	37.22 a	15.0	0.18	39.4	9.00 b	0.6 b	4.6	2.6 a	64.27 a
**LSD 5%**	**0.020**	**4.33**	**9.23**	**1.42**	**NS**	**NS**	**2.57**	**NS**	**NS**	**NS**	**0.8**	**0.94**	**NS**	**0.20**	**2.82**
**T**_**1**_	0.341 a	128.43 a	163.11 a	24.05 a	6.76 bc	79.0 b	40.38 a	21.0 a	0.26 a	46.0 a	6.90 c	5.4 a	3.9 c	1.9 b	40.77 a
**T**_**2**_	0.336 a	117.65 bc	155.16 ab	23.70 a	6.59 c	85.0 a	33.99 bc	15.0 b	0.17 b	30.7 b	11.20 a	4.6 ab	5.6 a	2.5 a	32.77 bc
**T**_**3**_	0.253 b	116.13 c	128.12 c	17.84 b	7.36 ab	86.0 a	31.38 c	14.0 b	0.16 b	34.5 b	9.80 b	3.7 b	4.2 c	2.0 b	29.27 c
**T**_**4**_	0.271 b	122.71 ab	148.05 b	19.14 b	7.77 a	84.0 a	35.93 b	16.0 b	0.19 b	44.2 a	10.5 ab	4.1 b	4.9 b	2.7 a	35.36 b
**LSD 5%**	**0.028**	**6.13**	**13.06**	**2.01**	**0.70**	**2.3**	**3.64**	**0.023**	**0.035**	**5.4**	**1.19**	**1.33**	**0.39**	**0.28**	**4.00**
**W**_**1**_**T**_**1**_	0.303 cd	116.36	132.70 cd	21.38 cd	6.27	78.0	40.86 a	22.0	0.28	45.4	7.2	10.3 a	3.9	1.5	4.49 d
**W**_**2**_**T**_**1**_	0.379 a	140.50	193.51 a	26.72 a	7.26	81.0	39.90 ab	19.0	0.24	46.5	6.6	0.6 d	3.9	2.3	77.05 a
**W**_**1**_**T**_**2**_	0.328 bc	106.80	145.07 c	23.10 bc	6.37	83.0	34.09 c	17.0	0.20	29.4	11.6	8.6 ab	5.6	2.3	3.46 d
**W**_**2**_**T**_**2**_	0.345 ab	128.50	165.25 b	24.29 ab	6.81	87.0	33.89 c	13.0	0.14	31.9	10.9	0.5 d	5.5	2.6	62.08 b
**W**_**1**_**T**_**3**_	0.238 e	104.28	118.72 d	16.84 e	7.40	86.0	24.46 d	14.0	0.16	31.7	10.2	6.6 c	4.2	1.7	5.21 d
**W**_**2**_**T**_**3**_	0.267 de	127.99	137.52 c	18.84 de	7.32	86.0	38.29 abc	14.0	0.16	37.3	9.4	0.7 d	4.2	2.4	53.33 c
**W**_**1**_**T**_**4**_	0.273 de	112.76	150.13 bc	19.30 de	7.79	85.0	35.06 bc	15.0	0.18	46.4	11.8	7.5 bc	5.3	2.3	6.12 d
**W**_**2**_**T**_**4**_	0.269 de	132.65	145.97 c	18.98 de	7.74	84.0	36.80 abc	16.0	0.19	42.0	9.1	0.7 d	4.6	3.0	64.60 b
**LSD 5%**	**0.040**	**NS**	**18.47**	**2.85**	**NS**	**NS**	**5.15**	**NS**	**NS**	**NS**	**NS**	**1.88**	**NS**	**NS**	**5.65**

NS = non-significant, W_1_ = *Physalis angulata*, W_2_ = *Physalis philadelphica*, T_1_ = Clay-1, T_2_ = Clay-2, T_3_ = Sandy loam, T_4_ = Silt clay loam, RGR = relative growth rate, PH = plant height, TFM = total fresh mass, TDM = total dry mass, FDR = fresh mass:dry mass ratio, SMF = shoot mass fraction, RL = root length, RMF = root mass fraction, RSR = root:shoot ratio, K = potassium, Mg = magnesium, Na = sodium, Ca = calcium, P = phosphorus, K/Na = potassium:sodium ratio, The values following different letters in a column are significantly different from each other at *p* ≤ 0.05

The interactive effects of weeds and soil textures were non-significant except RGR, FM, TDM and RL (Table 3). The higher RGR, TFM and TDM were observed for *P*. *philadelphica* on clay textured soils, while both weeds had similar values of these traits on remaining soils. Similarly, longer root system of both weeds was noted on clay textured soils (Table 9). Soil textures slightly affected the reproductive output, however, overall effect was non-significant (Figs 1 and 2). *P*. *philadelphica* produced the highest number of fruits and seeds per plant on all soil textures compared to *P*. *angulata* (Figs 1 and 2).

#### Nutrient uptake

Both weeds behaved differently for nutrient uptake except K and Ca on different types of soils (Table 5). *P*. *angulata* accrued higher amounts of Ca and Na while, *P*. *philadelphica* maintained higher K/Na ratio and accumulated more P (Table 9). The interactive effect of weeds and soil textures were non-significant except for Na and K/Na ratio (Table 5). *P*. *angulata* exhibited higher affinity for Na on all types of soils while, *P*. *philadelphica* maintained higher K/Na ratio like in other experiments of the study (Table 9).

Different growth and nutrient uptake traits exhibited significant positive and negative correlations among them (Table 10). RGR was positively correlated with PH, TFM, TDM, RMF, RSR and K/Na ratio, while had negative correlations with FDR, SMF and Na. Plant height was positively correlated with TFM, TDM, FDR, RL, K, P and K/Na ratio while, exhibited negative correlations with Mg, Na and Ca. TFM and TDM were negatively correlated with Na. The K/Na ratio was positively correlated with PH, TFM, TDM, K and P while, had negative correlation with Na and Mg (Table 10).

**Table 10:** Correlation between growth and nutrient uptake traits of two co-occurring invasive weeds grown on different soil types.

	RGR	PH	TFM	TDM	FDR	SMF	RL	RMF	RSR	K	Mg	Na	Ca	P	K/Na
**RGR**	1														
**PH**	.252*	1													
**TFM**	.709**	.570**	1												
**TDM**	1.00**	.252*	.709**	1											
**FDR**	-.539**	.341**	.155	-.539**	1										
**SMF**	-.292*	-.049	.020	-.292*	.369**	1									
**RL**	.216	.257*	.151	.216	-.075	-.392**	1								
**RMF**	.292*	.049	-.020	.292*	-.369**	-1.00**	.392**	1							
**RSR**	.292*	.049	-.020	.292*	-.369**	-1.00**	.392**	1.00**	1						
**K**	-.089	.571**	.357**	-.089	.559**	-.135	.295*	.135	.135	1					
**Mg**	-.228	-.352**	-.025	-.228	.202	.390**	-.238	-.390**	-.390**	-.129	1				
**Na**	-.359**	-.535**	-.387**	-.359**	.052	-.122	-.104	.122	.122	.144	.272*	1			
**Ca**	.015	-.342**	.055	.015	-.045	.269*	-.252*	-.269*	-.269*	-.340**	.810**	.036	1		
**P**	-.042	.503**	.374**	-.042	.413**	.256*	.172	-.256*	-.256*	.344**	.258*	-.379**	.271*	1	
**K/Na**	.270*	.745**	.475**	.270*	.180	.049	.227	-.049	-.049	.321**	-.376**	-.858**	-.227	.468**	1

* = Correlation is significant at *p* ≤ 0.05

** = Correlation is significant at *p* ≤ 0.01

RGR = relative growth rate, PH = plant height, TFM = total fresh mass, TDM = total dry mass, FDR = fresh mass:dry mass ratio, SMF = shoot mass fraction, RL = root length, RMF = root mass fraction, RSR = root:shoot ratio, K = potassium, Mg = magnesium, Na = sodium, Ca = calcium, P = phosphorus, K/Na = potassium:sodium ratio.

## Discussion

Successful seedling survival, vigorous growth, nutrient acquisition and fecundity are prerequisites of successful plant invasion [41]. These traits are discussed separately in the coming sections.

### Plant Survival

Seedling survival of both weeds was almost not affected by water stress (except severe water stress) and soil textures, whereas, salinity lowered the seedling survival percentage of both weeds (Table 2). *P*. *philadelphica* proved more tolerant compared to *P*. *angulata* under increasing water and salinity stress with higher survival percentage and longer survival time. Higher seedling mortality under increasing salinity is related either osmotic stress or ion toxicity [15]. Osmotic stress caused by low external water potential, ion toxicity and disrupted nutrient uptake, transport and utilization are three major impacts exerted by salinity on plant growth [42]. Higher accumulation of Na probably damaged the biological membranes and subcellular organelles resulting in abnormal growth and development which led to plant mortality [43, 44]. Since no mortality was observed for both tested weeds under water stress, seedling mortality in salinity experiments is thought to be the direct effect of ion toxicity rather than osmotic stress. The survival of *P*. *philadelphica* seedlings for longer period under high salinity levels seemed to be the result of lower Na accumulation and decreased leakage of K from the cell compared to *P*. *angulata* (Table 7). In the previous studies, lower transport of Na to shoots and higher selectivity of K over Na have been suggested as mechanism of salt tolerance [45]. The results of the present study indicate that both invasive weeds have evolved adaptive strategies to persist under prevailing environmental conditions of the invaded range. Blackburn et al. [46, 47], also indicated that higher survival rate of invasive plants under benign and harsh environments plays a vital role in range expansion and invasion success. As *P*. *philadelphica* survived for longer period, the results indicate that tested weeds differ in survival strategies under stressful environments. The preference of K over Na indicates that *P*. *philadelphica* can better withstand salinity than *P*. *angulata*.

Seedlings grown on different types of soils faced no adverse environmental conditions impacting water and nutrient uptake. Therefore, no mortality was observed on all types of soils. Higher survival rate is considered as a sign of sufficient resource availability rather than soil texture. Funk [41] indicated that e invasive plants recruit higher number of seedlings on resource rich soils regardless of texture.

### Growth and Fecundity

Vigorous growth, better resource acquisition, high fecundity along with higher plant survival are among the important traits promoting colonization, abundance and range expansion of invasive plants [46, 47]. However, these traits are repressed by critical factors such as water and nutrient availability, and soil salinity.

Impaired growth rate, decline in plant height, low biomass production, reduced reproductive output and disturbed nutrient balances are the general negative effects of abiotic stresses on plants [11, 15, 48, 49]. Both growth and fecundity are positively associated with high growth rate (or relative growth rate) which is considered as an influential trait in invasion ecology [50]. The growth rate, plant height, biomass production, nutrient acquisition and fecundity were evidently affected by abiotic stresses and relatively less altered by different soil textures in the current study. Higher RGR and TDM were noted for *P*. *philadelphica* under no and moderate water and salinity stresses. Whereas, increasing water deficit and salinity significantly impaired these traits of both weeds. Both water and salinity stresses decrease the moisture and nutrient availability to the growing plants through developing osmotic stress or ion toxicity [11, 15]. Therefore, decreased growth of the tested weeds under increasing water and salinity stresses are probably the results of lower water and nutrient uptake due to osmotic stress or ion toxicity. Moreover, salinity and drought pose negative effects on several physiological processes such as photosynthesis, respiration, starch metabolism, and nitrogen fixation resulting in meager plant growth [42]. The imposed stresses probably negatively affected these physiological processes in both weeds resulting in impaired growth.

Biomass allocation patterns were changed in both weeds under increasing water deficit and biomass allocation was increased to roots and decreased towards shoots. The change in biomass allocation patterns to roots and shoots is the direct effect of increasing water deficit as plants allocate more biomass where the resources are more limited [51, 52]. Moreover, lower moisture availability restricted root growth hence, plants allocated more resources towards shoots instead of roots. Contrastingly, decreased biomass allocation towards roots under increasing salinity is result of ion toxicity [15]. Therefore, change in biomass allocation patterns is considered as an important adaptive trait under adverse environmental conditions. The plasticity in biomass allocation of the plants in response to prevailing environmental stresses might be responsible for their improved tolerance.

Plants develop diverse morphological and physiological mechanisms to alleviate the negative effects of water and salinity stress [11, 15, 53]. Restriction of salt uptake, control of salt transport to shoots, extrusion of the accumulated salts from shoots and maintenance of higher K/Na ratio are some of the mechanisms involved in salt tolerance of plants [54]. Changing root-to-shoot ratio is considered as one of the mechanisms involved in the adaptations of plants to water deficit [55] however, root growth is generally less affected by water stress than shoot growth [56]. Increasing water deficit and salinity decreased root length and biomass production for both *Physalis* species. Increasing water deficit makes the soil compacted and offers hurdles in root penetration while excessive amounts of Na and Cl in the root zone also negatively affect the root growth. The decline in root length and biomass production in the current study are thought to be the result of mechanical impedance offered by the soil. The reduction in root length and biomass induced by drought and salinity has been reported for a number of different weeds [31, 32, 33, 34].

Soils with varying particle size provide highly variable amounts of water and nutrients due to differences in total specific surface areas. The edaphic diversity of a given area can restrict plant invasion. Soils with high clay and organic matter contents provide higher surface area for nutrient exchange and hold more water [20]. Both weeds had almost similar growth on different types of soils except clay. The better growth performance of both weeds on clay textured soil is linked with the better resource availability of clay soils compared to other textures included in the current study.

Overall, *P*. *philadelphica* produced higher number of seeds than *P*. *angulata* under all experimental conditions. Increasing water deficit and salinity lowered seed production while, different soil textures had no effect. Lower reproductive output under increasing severity of stresses is directly linked with impaired growth and nutrient acquisition because of low moisture availability and weak root system. Previous studies also revealed that increasing water stress lowered fecundity of different weeds [31, 32, 33]. The reduction in reproductive output of different populations of *P*. *angulata* under moderate and high water deficits and resource poor soils has also been reported by Travlos [27].

Osmotic adjustments, antioxidant defense system, CO_2_ exchange and change in photosynthesis and respiration are other important mechanisms opted by the plants to persist under stressful environments [42]. However, these mechanisms have not been explored in the current study. Descriptive studies on plant responses to abiotic stresses provide valuable insights for future research aiming at understanding the biological mechanisms of stress tolerance. Furthermore, no literature exist regarding effect of abiotic stresses and soil types on growth and development of the tested weeds. Therefore, current study reports interesting findings which necessitate the exploration of biological mechanisms behind enhanced tolerance of both weeds. Besides different populations arising from different climatic/ecological regions can be used in the future studies to explore the mechanisms imparting stress tolerance under ambient and changing environmental conditions.

### Nutrient Uptake

The abiotic stresses, salinity in particular, disturb the uptake and transportation of essential mineral nutrients [11, 15]. Water deficiency also negatively affects nutrient uptake by roots due to low moisture availability [57]. The lower transpiration rate, mechanical impedance of soil and ion toxicity are possibly responsible for lower nutrient uptake under water and salinity stress. Low transpiration rate reduces the nutrient transport from roots to shoots, imbalances active transport and membrane permeability, resulting in a reduced absorption power in the roots [11, 57, 58].

The decreased uptake of K ions under increasing water stress is the result of a weaker root system and lower available water in the root zone (Table 7), which lead to stomatal closure in plants and reduces transpiration [59]. Both weeds differed in K uptake under increasing water stress (Table 3). *P*. *angulata* improved K uptake with increasing water deficit, though the plant accumulated more Na and lowered K/Na ratio. The improved K uptake ensures better plant growth and imparts tolerance against adverse environmental conditions. A number of physiological processes such as enzyme activation, protein synthesis, photosynthesis, osmoregulation, cell extension, stomatal movement are governed by K in plants [11, 20]. The improved drought resistance of plants by application of K has also been reported by several researchers [60, 61].

Salinity negatively affected K uptake due to its competition with Na ions however, both weeds presented varying response (Table 7). *P*. *philadelphica* accumulated half amount of Na compared to *P*. *angulata* indicating that *P*. *philadelphica* employed an avoidance strategy from Na ions by preferring K in contrast to *P*. *angulata*. Lower Na uptake resulted in lesser osmotic stress and ion toxicity while higher K acquisition. Moreover, high biomass production of *P*. *philadelphica* is related to the maintenance of a higher K/Na ratio under all salinity levels. The results of current study are in accordance with several earlier reports [62, 63]. These researchers reported a linear relationship between higher biomass production and K/Na ratio in different plants as observed in the current study (Table 8).

Elevated level of Na ions in the soil result in decreased Ca and K uptakes, which results in K, Ca, and Mg ion imbalances [64]. Hussain et al. [65] also reported that rising salinity concentration decreased Ca and Mg uptake. Moreover, reduction in Ca uptake under higher Na accumulation affects several vegetative and reproductive functions of plants [61, 66]. In the current study, although K uptake was decreased with increasing soil salinity, the concentrations of Ca and Mg were also increased with increasing Na uptake (Table 5). *P*. *philadelphica* accumulated higher amounts of Ca and Mg ions compared to *P*. *angulata*. These interesting findings suggest that *P*. *philadelphica* has evolved the strategy of more Ca and Mg uptake along with the maintenance of a high K/Na ratio to avoid the nutrient imbalances enabling the plant to better tolerate salinity than *P*. *angulata*. The increased Ca and Mg uptake probably resulted in improved chlorophyll contents [67]. The improved chlorophyll content lead to higher photosynthesis and ultimately better plant growth.

There were slight differences in nutrient uptake between weeds on different types of soils. However, like in drought and salinity experiments, *P*. *angulata* showed higher affinity for Na compared to its co-occurring *P*. *philadelphica* (Table 7). High Na uptake develops osmotic stress, thus restricting moisture availability to growing plants. *P*. *philadelphica* accumulated higher amounts of K and maintained higher K/Na ratio. Both weeds exhibited higher nutrient uptake on clay soil. Better nutrient acquisition on clay textured soils is linked with improved solute transport from soil to roots because of higher available moisture. Since solute transport depends on soil moisture, hydraulic conductivity and the tortuosity factor, which are functions of soil texture [68]. The clay textures provide higher moisture and solute transport, therefore better performance of both weeds on clay textured soils is the possible result of these inherent characteristics of clay soils.

## Conclusions

It is concluded that both weeds have sufficient potential to expand their invasion range under current climate and can successfully adapt to increasing water deficit and soil salinity, and different soil textures. These adaptations will lead to higher abundance and range expansion of both plants in irrigated semi-arid and arid regions of the country. However, the tested invasive weeds behaved differently to water and salinity stresses, which suggest that dominance of both weeds will depend on prevailing environmental conditions. For example, *P*. *angulata* may dominate under increasing aridity however, detailed studies are needed to infer the actual adaptive mechanisms as K/Na ratio was decreased. Whereas, irrigation induced soil salinity could result in better adaptations and further range expansion of *P*. *philadelphica*.

Prediction of potential distribution areas for both weeds through incorporating the current results is needed to devise an early warning and rapid response system. Moreover, as the plants showed interesting strategies to cope with salinity and water stresses, detailed physiological and molecular studies can produce valuable insights to understand the tolerance mechanisms of these plants.
