# Correction: Invasion Potential of Two Tropical *Physalis* Species in Arid and Semi-Arid Climates: Effect of Water-Salinity Stress and Soil Types on Growth and Fecundity

**DOI:** 10.1371/journal.pone.0316419

**Published:** 2024-12-19

**Authors:** Cumali Ozaslan, Shahid Farooq, Huseyin Onen, Bekir Bukun, Selcuk Ozcan, Hikmet Gunal

The error bars were inadvertently omitted from Fig 2, and this error is rectified in the updated figure provided with this notice.

In addition, contrary to this article’s [1] Data Availability statement, the individual-level data underlying the published results were not provided with the manuscript. The first author provided the original data upon editorial request and these data have been made available in S1 File provided with this notice.

The underlying data and the corresponding methods and results have been reviewed by an independent member of the *PLOS ONE* Editorial Board. The Editorial Board member stated that the underlying data appear to support the overall published results, but commented that the method section of [1] includes an incorrect description of survival %. The first author acknowledges the error and clarifies that the accurate formula for determining the survival rate is the following:

$$Survival\;\%=\frac{Number\;of\;surviving\;plants}{Total\;number\;of\;transplanted\;plants}\times 100$$

## Supporting information

S1 File Individual-level data underlying the published results. (XLSX)
